# Denosumab versus zoledronic acid for osteoporosis treatment in patients with primary biliary cholangitis (the DELTA Study): A multicenter, non-inferiority randomized trial

**DOI:** 10.1097/HC9.0000000000000827

**Published:** 2025-10-07

**Authors:** Yoshitaka Arase, Tomomi Okubo, Taeang Arai, Masanori Abe, Tadashi Namisaki, Haruki Uojima, Kosuke Matsumoto, Keisuke Kakisaka, Toru Setsu, Yusuke Mishima, Kota Tsuruya, Shunji Hirose, Ryuzo Deguchi, Koichi Shiraishi, Masanori Atsukawa, Tadashi Ikegami, Akira Honda, Shuji Terai, Hitoshi Yoshiji, Atsumasa Komori, Atsushi Tanaka, Tatehiro Kagawa

**Affiliations:** 1 Division of Gastroenterology and Hepatology, Department of Internal Medicine, Tokai University School of Medicine, Kanagawa, Japan; 2 Division of Gastroenterology, Nippon Medical School Chiba Hokusoh Hospital, Inzai, Japan; 3 Division of Gastroenterology and Hepatology, Nippon Medical School, Tokyo, Japan; 4 Department of Community and General Medicine, Ehime University Graduate School of Medicine, Ehime, Japan; 5 Department of Gastroenterology, Nara Medical University, Nara, Japan; 6 Department of Gastroenterology, Kitasato University School of Medicine, Sagamihara, Japan; 7 Department of Medicine, Teikyo University School of Medicine, Tokyo, Japan; 8 Department of Gastroenterology and Hepatology, Iwate Medical University, Iwate, Japan; 9 Division of Gastroenterology and Hepatology, Niigata University Graduate School of Medical and Dental Sciences, Niigata, Japan; 10 Fujita Health University Haneda Clinic, Tokyo, Japan; 11 Wellaging Clinic, Tokyo, Japan; 12 Division of Gastroenterology and Hepatology, Department of Internal Medicine, Tokyo Medical University, Ibaraki Medical Center, Ibaraki, Japan; 13 Clinical Research Center, National Hospital Organization (NHO) Nagasaki Medical Center, Nagasaki, Japan

**Keywords:** adverse event, ALP, bisphosphonate, bone mineral density, receptor activator of nuclear factor kappa-B ligand

## Abstract

**Background:**

Osteoporosis is a common complication in patients with primary biliary cholangitis (PBC). This study aimed to compare the efficacy and safety of denosumab and zoledronic acid (ZOL) in treating osteoporosis in PBC patients.

**Methods:**

This multicenter, randomized, open-label trial enrolled Japanese patients with PBC and osteoporosis. Patients were randomized to receive either subcutaneous denosumab 60 mg every 6 months (denosumab group) or i.v. zoledronic acid 5 mg yearly (ZOL group). The primary endpoint was the mean percent change in bone mineral density (BMD) at the lumbar spine and total hip from baseline to 12 months.

**Results:**

Of 47 enrolled patients, 41 (87.2%) completed the study (denosumab: n=21; ZOL: n=20). At 12 months, lumbar spine BMD increased by 7.5% in the denosumab group and 6.4% in the ZOL group, demonstrating the non-inferiority of denosumab (95% CI: −1.6% to 3.8%). Although the total hip BMD increased more in the denosumab group than in the ZOL group (5.0% vs. 2.6%, *p*<0.01), the difference did not meet the predefined non-inferiority margin (95% CI: −1.3% to 6.2%). Serum ALP to upper limit of normal ratio and bone turnover markers significantly decreased in both groups; however, the rates of change were not significantly different between them. The incidence of adverse events was significantly lower in the denosumab group compared with the ZOL group (14.3% vs. 50.0%, *p*=0.013).

**Conclusions:**

Denosumab is a safe and effective treatment option for osteoporosis in patients with PBC.

## Graphical abstract

**Figure:**
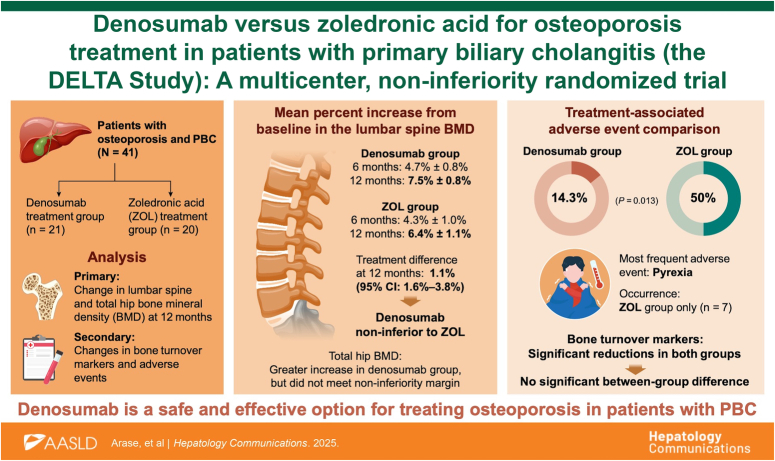

## INTRODUCTION

Primary biliary cholangitis (PBC) is an autoimmune cholestatic disease that predominantly occurs in middle-aged women; it is characterized by the progressive destruction of bile ducts, leading to fibrosis and liver failure when left untreated.^1^^,^^2^ In addition to pruritus and fatigue, osteoporosis is a major complication in patients with PBC.^3^ The development mechanisms of metabolic bone diseases in patients with PBC are complicated, involving the defective absorption of fat-soluble vitamins and the direct effect of cholestasis on bone metabolism.^4^ In a recent meta-analysis, patients with PBC had a relative risk of 2.79 for osteoporosis and an OR of 1.86 for developing bone fractures compared with patients without PBC.^5^ Moreover, a large population-based cohort study indicated that the risk of fracture and rate of post-fracture mortality in patients with PBC were significantly higher than those in the matched controls in the general population.^6^

The effectiveness of bisphosphonate therapy for osteoporosis in patients with PBC has been reported. Zein et al.^7^ reported that alendronate significantly increased lumbar spine bone mineral density (BMD) by 10.4% compared with placebo (−0.12%) in patients with PBC-related osteoporosis. A recent randomized clinical trial showed that weekly alendronate and monthly ibandronate therapy increased BMD in patients with PBC; however, adherence was significantly better in the ibandronate group.^8^ Bisphosphonates are generally well tolerated; however, they are not recommended for patients with severe renal impairment or hypocalcemia. Moreover, they can cause adverse effects such as esophageal injury and osteonecrosis of the jaw.

Receptor activator of nuclear factor kappa-B ligand (RANKL) is known as a tumor necrosis factor-related activation-induced cytokine, and it is expressed in various cells, including osteoblasts. It binds with receptor RANK on osteoclast and precursor cell surfaces, promoting the differentiation and activation of osteoclasts involved in bone resorption.^9^ Denosumab is a human monoclonal antibody that inhibits RANKL, a cytokine essential for osteoclast formation and function.^10^ Reportedly, subcutaneous denosumab treatment increased BMD by blocking the binding of RANKL to RANK and inhibiting the activity of osteoclasts. This suppressed bone resorption and reduced the risk of vertebral, nonvertebral, and hip fractures in postmenopausal women with osteoporosis.^11^ Denosumab is effective and safe in treating osteoporosis even in patients with chronic kidney disease, including those undergoing dialysis.^12^^–^^14^ Denosumab has different mechanisms of action and can be used as a first-line treatment or as an alternative to bisphosphonates. Recently, we reported that short-term (1-year) and long-term (3-year) treatment with denosumab significantly increased BMD without adverse events in patients with autoimmune liver diseases, including those with PBC.^15^^,^^16^ Saeki et al.^17^ reported that denosumab is a safe and beneficial treatment option for osteoporosis in patients with chronic liver disease.

To the best of our knowledge, no study has compared denosumab and bisphosphonates for PBC-related osteoporosis. In this randomized trial, we aimed to compare the efficacy and safety of denosumab and zoledronic acid (ZOL) for osteoporosis in patients with PBC.

## METHODS

### Study design and patient population

This multicenter, randomized, open-label, parallel-group trial (*DE*nosumab versus zo*L*edronic acid for os*T*eoporosis in p*A*tients with PBC: the DELTA Study) was conducted from May 2018 to March 2022 in 10 centers in Japan. Ethical approval for this study was provided by the Institutional Review Board for Clinical Research of Tokai University School of Medicine (approval no. 17R-64) and the local institutional review board at each participating institution. All research was conducted in accordance with the ethical standards of the institutional research committee and the 1964 Declaration of Helsinki and its later amendments and comparable ethical standards. All participants provided written consent. This study was registered at the University Hospital Medical Information Network Clinical Trials Registry (number UMIN000031384).

The inclusion criteria were as follows: (1) patients diagnosed with PBC based on the PBC clinical practice guidelines edited by the Ministry of Health, Labor and Welfare in Japan^18^; (2) patients diagnosed with osteoporosis according to the World Health Organization criteria (T score ≤−2.5)^19^; (3) women or men aged ≥20 years and ≤89 years; and (4) patients who had not previously received any anti-osteoporotic treatment, including denosumab and bisphosphonates. The exclusion criteria were as follows: (1) patients with hypocalcemia (defined as serum calcium concentration <8.5 mg/dL); (2) patients with estimated glomerular filtration rate <35 mL/min/1.73 m^2^; (3) patients with a history of glucocorticoid use; and (4) patients with a requirement of dental treatment.

Participants were randomly assigned at a 1:1 ratio to receive either subcutaneous denosumab 60 mg every 6 months or 15-minute i.v. ZOL 5 mg once for 12 months. Randomization was stratified according to 5-year age groups and sex. Stratified randomization was performed by computer allocation conducted by Viedoc Japan Co., Ltd (Tokyo, Japan). All patients were administered 0.75 μg/day eldecalcitol throughout the study period. The severity of adverse events was assessed according to the Common Terminology Criteria for Adverse Events version 5.0.^20^

### Clinical and laboratory parameters

We collected baseline information such as age, sex, body mass index (BMI, calculated as weight in kg/height in m^2^), history of fragility fracture, concomitant metabolic disease, therapeutic drug, laboratory parameters, and BMD. Diagnosis of metabolic diseases was based on defined characteristics and patients’ medication regimen. Patients with fasting serum glucose level ≥100 mg/dL or 2-hour post-load glucose level ≥140 mg/dL or HbA1c ≥6.0% or patients taking glucose-lowering medication were diagnosed with type 2 diabetes mellitus. Blood samples were obtained in the outpatient clinic following a 12-hour overnight fast. Laboratory parameters included blood cell counts; levels of serum AST, ALT, ALP, GGT, total bilirubin, creatinine, calcium, 25-hydroxyvitamin D, intact parathyroid hormone, tartrate-resistant acid phosphatase 5b (TRACP-5b) as a marker of bone resorption, and bone-specific alkaline phosphatase (BAP) as a marker of bone formation; and estimated glomerular filtration rate. If the serum albumin concentration was <4 g/dL, corrected calcium concentration was calculated as follows: serum calcium concentration + (4.0—serum albumin concentration). If the serum albumin concentration was ≥4 g/dL, no additional calculations were performed. Hypocalcemia was diagnosed when the serum calcium concentration was <8.5 g/dL. BMD at the lumbar spine (L2–L4) and total hip was evaluated using dual-energy X-ray absorptiometry at baseline, 6 months, and 12 months. Osteoporosis was diagnosed according to the World Health Organization criteria: T score ≤−2.5 for osteoporosis, between −2.5 and −1.0 for osteopenia, and >−1.0 for normality.^19^ Vertebral fractures were evaluated using spinal lateral x-rays at baseline and 12 months. The new peripheral fractures were also recorded throughout the study.

To assess the serum calcium level and renal function, blood samples from both groups were evaluated 1−2 weeks after therapeutic intervention. Subsequently, patients were required to visit the outpatient clinic quarterly. They underwent physical examinations and laboratory blood tests on each visit. During this study, additional administration of drugs such as hormone replacement, selective estrogen receptor modulators, vitamin K, calcitonin, parathyroid hormone, and corticoids, which may affect treatment efficacy and safety, was prohibited. Patient enrollment, randomization, and data recording were managed using an Electronic Data Capture system provided by Viedoc Japan Co., Ltd (Tokyo, Japan).

### Study endpoints

The primary endpoint was the evaluation of the non-inferiority of denosumab to ZOL for the mean percent change in lumbar spine and total hip BMD between baseline and 12 months. The secondary endpoints were the time course changes in laboratory parameters and the incidence of adverse events.

### Statistical analysis

Demographic data are presented as mean±SD for continuous variables and as number (%) for categorical variables. The BMD change and treatment-related biomarker levels are presented as mean±SE. Changes within each group were evaluated using paired *t* tests and differences between the groups were examined using unpaired *t* tests.

We aimed to verify the non-inferiority of denosumab to ZOL in terms of the mean change in lumbar spine and hip BMD at 12 months. To derive the non-inferiority margins, we referred to the effect of ZOL treatment at 12 months on the lumbar spine and total hip BMD relative to that of placebo in a Japanese phase III trial involving postmenopausal women.^21^ The margins were 6.0% (95% CI: 4.89%–6.68%) for the lumbar spine and 2.5% (95% CI: 1.76%–2.92%) for the total hip. A 50% preservation of ZOL treatment effect was clinically meaningful, leading to non-inferiority margins of −2.4% for the lumbar spine and −0.9% for the total hip. If the lower limit of the 95% CI for the mean difference exceeds the non-inferiority margin, non-inferiority will not be established. Based on these assumptions, a total cohort size of 72 (36 patients per group) would provide a power of 90% with an α error of 2.5% (one-sided). This will identify a significant difference in the mean percent change in the lumbar spine BMD between the groups at 12 months. This cohort size achieved >80% power for the total hip BMD. To permit an estimated dropout rate of 10%, a total cohort size of 80 patients was projected.

All analyses were performed using SPSS version 26 (SPSS Japan, Tokyo, Japan), and results with *p* values <0.05 were considered statistically significant.

## RESULTS

### Patient characteristics

**FIGURE 1:**
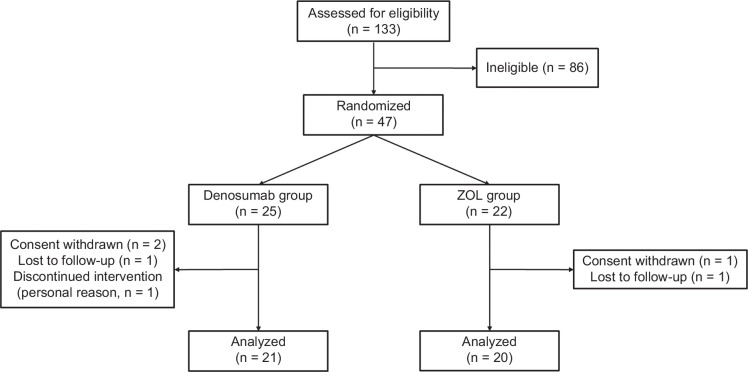
Flow diagram of patient selection in the present study. Abbreviation: ZOL, zoledronic acid.

In total, 47 patients were enrolled and randomly assigned to the denosumab (n=25) and ZOL (n=22) groups between May 2018 and March 2021. Of them, 41 patients (87.2%), 21 patients in the denosumab group and 20 patients in the ZOL group, completed the study (Figure 1). In the denosumab group, the reasons for discontinuation included withdrawal (2 patients), loss to follow-up (1 patient), and personal reasons (1 patient). In the ZOL group, one patient withdrew, while another was lost to follow-up. Due to the global outbreak of COVID-19, the progress of the trial was hindered, and it was ultimately terminated before reaching the target number of participants.

The characteristics of the patients who completed the trial are shown in Table 1. The average age of patients in the denosumab and ZOL groups was 68.0±9.3 and 69.0±8.3 years, respectively. The majority of the patients were women, with only one man in the denosumab group. At baseline, the groups were similar with respect to age, BMI, habits, previous fractures, complications, ursodeoxycholic acid or bezafibrate use, biochemical features, and BMD value.

**TABLE 1:** Clinical characteristics of patients

	Denosumab group (n=21)	ZOL group (n=20)	*p*
Age (y)	68.0±9.3	69.0±8.3	0.706
Female, n (%)	20 (95.2)	20 (100)	0.335
BMI (kg/m^2^)	21.5±2.7	21.0±3.3	0.584
History of fracture, n (%)	3 (14.3)	3 (15.0)	0.950
Current smoker, n (%)	4 (19.0)	2 (10.0)	0.423
Habitual drinking, n(%)	7 (33.3)	4 (20.0)	0.348
Complication			
Type 2 DM, n (%)	1 (4.8)	2 (10.0)	0.532
Dyslipidemia, n (%)	11 (52.4)	7 (35.0)	0.274
Sjögren syndrome, n (%)	0 (0)	1 (5.0)	0.311
Autoimmune thyroid disease, n (%)	0 (0)	1 (5.0)	0.311
Treatment			
UDCA, n (%)	21 (100)	19 (95.0)	0.311
Bezafibrate, n (%)	7 (33.3)	4 (20.0)	0.348
Albumin (g/dL)	3.9±0.4	4.2±0.3	0.064
Prothrombin ratio (%)	99.5±14.7	98.8±19.7	0.893
AST (IU/L)	32.7±18.9	29.3±10.5	0.476
ALT (IU/L)	24.1±16.3	18.0±8.0	0.138
ALP/ULN	1.21±0.94	1.06±0.55	0.615
GGT (IU/L)	89.2±95.4	55.9±53.2	0.177
Total bilirubin (mg/dL)	0.9±0.8	0.7±0.2	0.512
Creatinine (mg/dL)	0.7±0.2	0.7±0.1	0.706
eGFR (mL/min/1.73 m^2^)	69.7±17.6	64.3±10.5	0.246
Ca (mg/dL)	9.5±0.4	9.3±0.4	0.188
25(OH)D^a^ (ng/mL)	15.5±7.8	15.8±3.5	0.877
Intact PTH^b^ (pg/mL)	37.5±8.8	37.5±17.3	0.998
TRACP-5b^c^ (mU/dL)	336.7±140.2	354.8±181.1	0.724
BAP^d^ (μg/L)	17.7±10.6	17.1±10.7	0.863
Lumbar spine BMD (g/cm^2^)	0.77±0.13	0.71±0.08	0.064
Lumbar spine T score	−2.60±1.06	−2.94±1.06	0.090
Total hip BMD (g/cm^2^)	0.64±0.11	0.60±0.10	0.235
Total hip T score	−2.49±0.75	−2.54±0.79	0.854

*Note*: Data are expressed as mean±SD or number (%).

^a^ Normal range: ≧30 ng/mL.

^b^ 10–65 pg/mL.

^c^ 170–590 mU/dL (male) and 120–420 mU/dL (female).

^d^ 3.7–20.9 μg/L (male) and 3.8–22.6 μg/L (female).

Abbreviations: 25(OH)D, 25-hydroxyvitamin D; BAP, bone-specific alkaline phosphatase; BMD, bone mineral density; BMI, body mass index; Ca, calcium; DM, diabetes mellitus; eGFR, estimated glomerular filtration rate; PTH, parathyroid hormone; TRACP-5b, tartrate-resistant acid phosphatase 5b; UDCA, ursodeoxycholic acid; ULN, upper limit of normal; ZOL, zoledronic acid.

### Effects of denosumab and ZOL treatment on BMD

**FIGURE 2:**
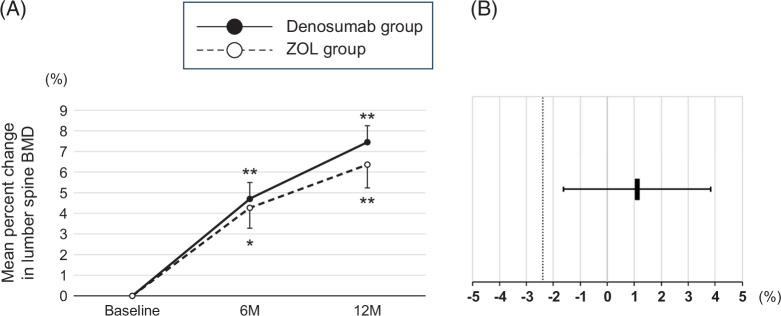
(A) Changes in the lumber spine BMD in the denosumab and ZOL groups at baseline and at 6 and 12 months of treatment. Data are shown as a mean±SE. Changes within each group were examined using paired *t* tests. *p<0.05 compared with baseline and **p<0.01 compared with baseline. (B) The point estimate of the mean difference in change rate in the lumbar spine BMD from baseline to 12 months between the denosumab and ZOL groups (denosumab group−ZOL group), and the 95% CI are shown. The dashed line indicates the non-inferiority margin. The point estimate was 1.1, and the lower bound of the 95% CI exceeded the non-inferiority margin, indicating that denosumab was non-inferior to ZOL. As the CI crossed 0, superiority was not demonstrated. Abbreviations: BMD, bone mineral density; ZOL, zoledronic acid.

The mean percent change from baseline in the lumbar spine BMD at 6 and 12 months in the denosumab group significantly increased by 4.7%±0.8% and 7.5%±0.8%, respectively. The mean percent change from baseline in the lumbar spine BMD at 6 and 12 months in the ZOL group also significantly increased by 4.3%±1.0% and 6.4%±1.1%, respectively (Figure 2A). The lumbar spine BMD at 12 months improved in all patients in the denosumab group, whereas in the ZOL group, 18 patients (90.0%) improved and 2 patients (10.0%) worsened. The treatment difference of 1.1% (95% CI: −1.6% to 3.8%) at 12 months in the lumbar spine BMD met the predefined non-inferiority margin of −2.4% (Figure 2B).

**FIGURE 3:**
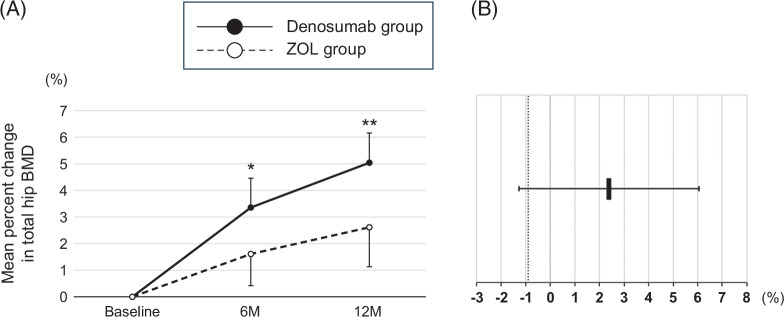
(A) Changes in the total hip BMD in the denosumab and ZOL groups at baseline and at 6 and 12 months of treatment. Data are shown as a mean±SE. Changes within each group were examined using paired *t* tests. *p<0.05 compared with baseline and **p<0.01 compared with baseline. (B) The point estimate of the mean difference in change rate in the total hip BMD from baseline to 12 months between the denosumab and ZOL groups (denosumab group−ZOL group), and the 95% CI is shown. The dashed line indicates the non-inferiority margin. The point estimate was 2.4, and the lower bound of the 95% CI was below the non-inferiority margin; thus, denosumab was not demonstrated to be non-inferior to ZOL. Abbreviations: BMD, bone mineral density; ZOL, zoledronic acid.

The mean percent change from baseline in the total hip BMD at 6 and 12 months in the denosumab group significantly increased by 3.4%±1.1% and 5.0%±1.1%, respectively, whereas that in the ZOL group increased by 1.6%±1.2% and 2.6%±1.5%, respectively, but neither was significant (Figure 3A). The total hip BMD at 12 months improved in 18 patients (85.7%) and 3 patients (14.3%) worsened in the denosumab group, whereas in the ZOL group, 17 patients (85.0%) improved and 3 patients (15.0%) worsened. The treatment difference of 2.4% (95% CI: −1.3% to 6.2%) in the total hip BMD at 12 months did not meet the predefined non-inferiority margin of −0.9% (Figure 3B).

### Effect of denosumab and ZOL treatment on bone turnover marker levels

**FIGURE 4:**
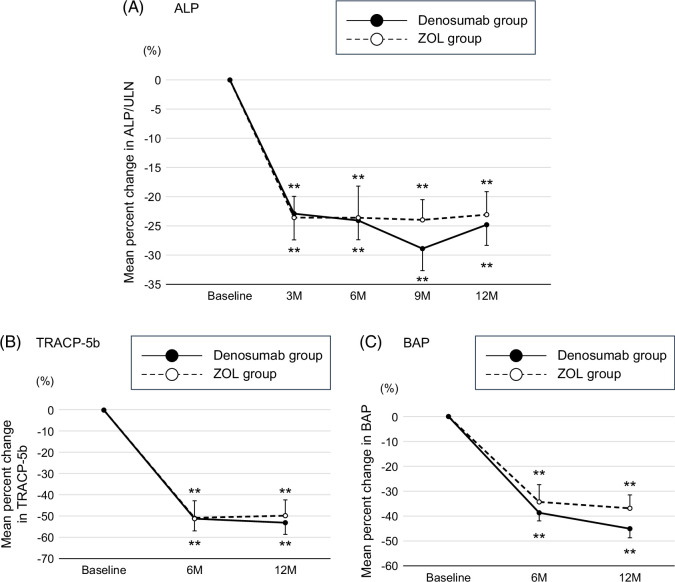
Changes in treatment-related biomarker levels in the denosumab and ZOL groups. (A) Ratio of serum ALP to ULN, (B) TRACP-5b, and (C) BAP. Data are shown as a mean±SE. Changes within each group were examined using paired *t* tests. *p<0.05 compared with baseline and **p<0.01 compared with baseline. Abbreviations: BAP, bone-specific alkaline phosphatase; TRACP-5b, tartrate-resistant acid phosphatase 5b; ULN, upper limit of normal level.

The changes in bone turnover marker levels are shown in Figure 3. The ratio of serum ALP to the upper limit of normal (ULN) level (ALP/ULN) significantly decreased by 22.9%±3.7% in the denosumab group (*p*<0.01) and 23.6%±4.5% in the ZOL group (*p*<0.01) at 3 months. These values were maintained at a similar level for 12 months in both groups (Figure 4A). The rate of change in the ALP/ULN at 12 months did not differ between the groups (−24.8%±4.0% vs. −23.1%±3.5%, *p*=0.75). The bone turnover marker levels also significantly decreased at 6 and 12 months in both groups; however, the rates of change did not differ between the groups at 12 months: TRACP-5b (−52.9%±5.5% vs. −49.6%±7.4%, *p*=0.73) and BAP (−45.0%±3.8% vs. −36.9%±5.5%, *p*=0.27) (Figures 4A, B).

### Safety

Adverse events that occurred during the study period were as follows: 7 cases of pyrexia; 4 cases of hypocalcemia; 2 cases of headache; and 1 case each of arthralgia, nausea, back pain, myalgia, and fracture. The most frequent adverse event was pyrexia, occurring exclusively in the ZOL group. The incidence of adverse events was significantly lower in the denosumab group than in the ZOL group: 14.3% versus 50.0% (*p*=0.013; Table 2). There was one fragility fracture in the ZOL group; a 54-year-old woman fell and broke her left ankle. According to the investigator, this fracture was not directly related to the treatment. There were no severe adverse events in both groups. In addition, fresh vertebral fractures and osteonecrosis of the jaw were not observed during the study.

**TABLE 2:** Incidence of adverse events

	Denosumab group (n=21)	ZOL group (n=20)	*p*
Patients with adverse events, n (%)	3 (14.3)	10 (50)	0.013
Adverse event			
Pyrexia	0 (0.0)	7 (35.0)	
Hypocalcemia	2 (9.5)	2 (10.0)	
Headache	0 (0.0)	2 (10.0)	
Arthralgia	1 (4.8)	0 (0.0)	
Nausea	0 (0.0)	1 (5.0)	
Back pain	0 (0.0)	1 (5.0)	
Myalgia	0 (0.0)	1 (5.0)	
Fracture	0 (0.0)	1 (5.0)	

*Note*: Data represent n (%).

Abbreviation: ZOL, zoledronic acid.

## DISCUSSION

In this study, we demonstrated that denosumab treatment was non-inferior to ZOL treatment in increasing BMD at the lumbar spine at 12 months and was associated with fewer adverse effects than ZOL in the treatment of osteoporosis in patients with PBC. However, this was an underpowered study, and the non-inferiority of denosumab to ZOL in increasing the total hip BMD was not confirmed. To the best of our knowledge, this is the first randomized trial comparing denosumab and bisphosphonates for osteoporosis in patients with PBC.

Osteoporosis is characterized by low bone mass and microarchitectural deterioration of bone tissue, leading to an increased risk of fragility fractures, decreased health-related quality of life, and ultimately poor survival.^22^ Osteoporosis is a common complication of PBC, and its prevalence in patients with PBC reportedly ranges from 20% to 52%.^23^^–^^25^ Regarding changes in BMD, the average annual change rate is −1.1% for healthy women, −2.3% for patients with liver cirrhosis, and −3.3% for patients with PBC.^26^ These figures indicate that patients with PBC are more susceptible to osteoporosis. Therefore, it is recommended that all patients with PBC should be evaluated for BMD.^1^^,^^18^

In this study, the mean increase in BMD after 12 months of denosumab therapy was 7.5% for the lumbar spine and 5.0% for the total hip, which aligned with previous reports in Japanese patients with osteoporosis.^27^ Non-inferiority of denosumab to ZOL was observed at the lumbar spine, but not at the total hip. The total hip BMD increased significantly in the denosumab group; however, non-inferiority to ZOL was not observed. There was also a significant variability in the effectiveness of the total hip. A recent meta-analysis revealed that the total hip is the most sensitive predictor for reduced risk of vertebral, nonvertebral, and proximal femoral fractures following osteoporosis treatment.^28^ It has been reported that if the BMD of the total hip could be increased by 3.18%, fractures could be prevented at all sites.^28^ In this study, only the denosumab group achieved an increase in BMD of 5.0% or more at the total hip at 12 months. Thus, the use of denosumab could prevent future fractures in patients with PBC.

In osteoporosis treatment, observing changes in bone metabolic marker levels is important to determine treatment effectiveness. TRACP-5b and BAP were used in this study because they are excellent bone turnover markers with minimal diurnal variation and are rarely affected by diet or renal function. In both groups, TRACP-5b and BAP rapidly decreased 6 months after treatment. This decrease was maintained even at 12 months; however, no statistical difference was observed in the degree of decrease between the groups.

In this study, the serum ALP level significantly decreased at 12 months of treatment in both groups. This was similar to the results of a randomized clinical trial of denosumab and ZOL in patients with postmenopausal osteoporosis.^29^ During the study period, there were no additions or changes in PBC treatment, such as ursodeoxycholic acid or bezafibrate, in any of the patients. The decrease in the serum ALP levels presumably reflects the suppression of excessive bone turnover,^30^ indicating the favorable effects of both drugs. Conversely, serum ALP level reduction is a surrogate marker of treatment efficacy in PBC. In PBC, the RANK–RANKL axis may also have implications beyond osteoclastogenesis. A study demonstrated that the expression of RANK and RANKL in cholangiocytes around bile ducts was significantly greater in patients with PBC than in those with other liver diseases,^31^ suggesting the involvement of the RANK–RANKL axis in the mechanism of bile duct injury in PBC. Therefore, denosumab may improve bone metabolism and liver function in this intractable disease. In this study, we carefully analyzed the changes in ALP level between the groups; however, we found no difference in the decrease rate. The serum ALP has 3 isoenzymes: hepatobiliary system-related, bone-related, and intestinal epithelium-related ALP. In our previous study, administering denosumab to 6 patients with PBC significantly reduced the ALP level; however, it was largely attributable to the decline in bone-related isozyme level.^15^^,^^16^ As the ALP isoenzymes were not measured in this study, the hypothesis that the RANK–RANKL axis is involved in bile duct injury in PBC requires further investigation.^32^

Regarding adverse events, the denosumab group had fewer adverse events than the ZOL group. The most notable difference in the adverse events was the occurrence of pyrexia in 35.0% of patients in the ZOL group. This finding aligns with the results of the ZONE study involving Japanese postmenopausal women, where 39.3% of participants reported pyrexia.^21^ When ZOL is first administered, flu-like symptoms, including pyrexia, myalgia, and headache, may occur a few days after administration. This is called an acute phase reaction.^33^ Regarding the frequency of the acute phase reaction, the HORIZON study, a large-scale study including non-Japanese individuals, showed a low rate of 16.1%,^34^ which may reflect ethnic differences in the acute phase reaction to i.v. administration of bisphosphonates.^35^ Most of the acute phase reactions occurring in this study resolved within 3 days after onset, as previously reported.^36^ A typical adverse event of bone-resorbing drugs is hypocalcemia; however, in this study, its incidence was low in both groups; furthermore, all adverse events were asymptomatic and mild.

This study had some limitations. First, the target number of participants was not reached. The trial was discontinued before achieving the target enrollment, partly due to the outbreak of COVID-19 and safety concerns associated with zoledronic acid. Second, this study was a short-term analysis lasting just 12 months. Hence, we could not clarify the long-term outcomes (such as fragility fracture and adverse events, including osteonecrosis of the jaw). Finally, regarding the primary endpoint, an intention-to-treat analysis was not feasible due to the absence of data on BMD after administration in patients who withdrew consent or were lost to follow-up. However, for secondary endpoints, including laboratory parameters and adverse events, an intention-to-treat analysis was conducted using data from 22 patients in the denosumab group (including one who discontinued intervention) and 20 patients in the ZOL group, and the results of this analysis were largely consistent with those of the per-protocol analysis.

## CONCLUSIONS

In this multicenter, randomized trial of denosumab treatment every 6 months and ZOL treatment once yearly in patients with PBC, denosumab was non-inferior to ZOL in increasing BMD at the lumbar spine over 12 months, with fewer adverse effects than ZOL. Denosumab is an effective therapeutic agent for osteoporosis in patients with PBC. Further long-term studies are needed to assess fracture prevention in PBC-related osteoporosis.
